# Effect of Low Corrosion Levels on the Bond Performance of Lap-Splices in Reinforced Concrete Beams

**DOI:** 10.3390/ma18235300

**Published:** 2025-11-24

**Authors:** Agha Syed Muhammad Gillani, Chongku Yi, Kee-Jeung Hong

**Affiliations:** 1 School of Civil and Environmental Engineering, Kookmin University, Seoul 02707, Republic of Korea; aghasmg@gmail.com; 2 School of Civil, Environmental and Architectural Engineering, Korea University, Seoul 02841, Republic of Korea; chongku@korea.ac.kr

**Keywords:** reinforced concrete, lap-splice, corrosion, bonding, beam

## Abstract

The Korean Highway Bridge Design Code introduced requirements for sufficient tensile lap-splice lengths in 2005. Bridge piers designed before 2005 might not have enough tensile lap-splice length. In addition to that, old structures are susceptible to corrosion due to several environmental factors. Thus, to investigate the effect of lap-splice lengths on the bending behavior of the old piers, twelve reinforced concrete (RC) beams were fabricated with sufficient and insufficient tensile lap-splice length, electrochemically corroded and tested under bending. Six of these beams, with sufficient lap-splice lengths, failed in a ductile manner after reinforcement bar (rebar) yielding occurred, whereas the remaining six, with insufficient lap-splice lengths, failed in a brittle manner owing to lap-splice bond failure before rebar yielding. The tested low-corrosion (< 4% by mass) beams exhibited significantly higher load-carrying capacities than non-corroded counterparts tested in our previous study. These results correlated with previously reported results by other researchers, indicating that low corrosion levels do not critically compromise the flexural performance of RC piers. Nevertheless, piers featuring insufficient lap-splice lengths must be rehabilitated to ensure seismically sufficient ductile flexural behavior.

## 1. Introduction

In Korea, seismic loads were introduced in 1987 [1], with prescriptive recommendations for sufficient tensile lap-splice lengths following in the Korean Highway Bridge Design Code in 2005 [2]. Consequently, structural members, such as bridge piers, designed before 2005 might not have sufficient tensile lap-splice lengths, rendering them potentially vulnerable to seismic loads. In addition to this design deficiency, these aging structures are highly susceptible to environmental corrosion at the lap-splice interface, which can severely compromise their bond strength and ductility. Thus, it is necessary to investigate the seismic performance of existing corroded piers with insufficient lap-splice length.

During earthquakes, relatively tall bridge piers primarily function as flexural members against lateral loads. However, testing full-scale bridge piers entails high costs related to fabrication, corrosion setup, and experimental loading. Therefore, we tested small-scale RC beams instead of actual (real-size) piers under bending. This approach is a justified study because the beams accurately replicate the flexural behavior observed in real-size pier structures. We acknowledge that the effect of axial forces, which are present in piers but were not applied in these beam tests, should be explored in future studies.

Reinforcement corrosion adversely affects the strength and serviceability of RC members. The inherent high alkalinity of the pore solution of the concrete (pH 13–14) forms a passive layer around the reinforcement, thereby protecting the rebars from corrosion [3]. However, the exposure of RC members to aggressive agents, such as chloride ions (such as seawater and deicing salts) and acidic gases, counteracts this alkalinity, critically lowering the pH. Extant studies explored the factors affecting RC corrosion levels (e.g., pore saturation, humidity, and cement chloride content) [4,5,6,7,8,9], the effects of corrosion on the bonds around single reinforcement bars (rebars) [10,11], and the residual load-carrying capacities of RC structures [12,13,14]. This structural deterioration is physically driven by the oxidation of rebars, which is mainly caused by the carbonation of the concrete cover and chloride ion penetration, leading to the formation of rust particles on the surface of rebars [15]. These rust particles can expand to several times the volume of the original oxidized rebars, creating immense internal tensile stress that causes splitting cracks and subsequent deterioration of the concrete cover [16,17,18,19,20]. Furthermore, corrosion-induced depletion of the rebar cross-section decreases the mechanical interlock between rebar and concrete, thereby critically lowering the load-carrying capacities and bond strengths of RC members [21,22,23,24]. Notably, this effect is more prominent in plain bars than in deformed ones [25].

Rebars exhibit two primary corrosion types: “general (or uniform) corrosion” and “pitting (or local) corrosion” [26]. General corrosion affects a substantial rebar surface area, resulting in visible rust stains, cracking, and deterioration of the concrete cover. Conversely, pitting corrosion may not cause significant surface staining or disruption of concrete cover, complicating its assessment during structural inspection [27]. Studies have demonstrated the behavior of naturally corroded structural members through two primary approaches: laboratory exposure to chloride environments under service loads, or by extracting specimens directly from corrosion-damaged actual structures [28,29,30,31,32]. However, this approach requires access to specimens subjected to prolonged natural or controlled corrosion, often spanning over a decade. To achieve time efficiency, most studies on the effects of corrosion on reinforced concrete utilized accelerated corrosion methods, e.g., the application of impressed current and CaCl_2_ admixtures [33,34,35,36,37,38]. In this study, we employed an impressed current technique along with weekly wetting–drying cycles (4 days of wetting and 3 days of drying), to get corrosion similar to the natural corrosion process as done in previous studies [21].

Earlier studies demonstrate that low corrosion levels can increase the bond strength of specimens [19,39]. However, the critical corrosion level (defined as the threshold after which bond performance begins to decline) has been established by earlier researchers across a wide range (0.4–4%) [10,40,41]. There are generally three categories of tests that can be used to assess bond strength: “pullout or beam-end tests,” “tension-stiffening tests,” and “beam tests.” However, most studies examining corrosion effects utilized pullout or beam-end tests, where the rebars and concrete are in tension and compression, respectively [17,22,24,42,43,44]. However, the stress conditions in these tests do not accurately replicate real-world structural stress conditions, where concrete and rebar are in tension or compression simultaneously. Some studies utilized tension-stiffening tests, which simulate simultaneous tension in rebars and concrete, to investigate corrosion effects on bond strength [45,46,47,48]. However, relatively fewer studies are available on corroded beam tests [40,41,49].

Lap-splices are unavoidable in RC structural members because rebars must be cut for delivery and installation. Along the lap-splice length, stress increases from zero at the unloaded end to the maximum at the loaded end. Existing piers designed before 2005 often feature lap-splices that fail to meet current code requirements for adequate ductility. In addition to this structural vulnerability, corrosion occurring at the lap-splice location can incur an additional, critical loss of ductility [50]. However, only a very few experimental studies have explored the effect of corrosion on the bond behavior of lap-spliced RC beams [51,52,53]. Shihata studied the behavior of RC beams exhibiting insufficient lap-splice lengths and observed a significant loss of bond strength in corroded specimens [51]. Pantazopoulou et al. studied the behaviors of 22 RC beams with various corrosion levels (1.29–16.62%) and lap-splice lengths (five to forty-five times the diameter of tensile rebars) [52]. They demonstrated that shorter corroded lap-splices had a significant reduction in bond strength and failed in a brittle manner without the rebars yielding. Conversely, longer corroded lap-splices exhibited significant post-yielding deformation and failed in a ductile manner. However, the specimens in these studies lacked stirrups in the lap-splice region; hence, they did not consider the effect of the confinement provided by stirrups on the bond performance of corroded lap-spliced beams. Moodi et al. studied 20 beams featuring insufficient lap-splice lengths (200 mm) and different numbers of epoxy-coated stirrups (0–3) within the lap-splice region [53]. They observed that an increased number of stirrups significantly enhanced the bond strength of corroded lap-splices and improved the ductility and energy dissipation of RC beams. However, the stirrups in that study were protected from corrosion using epoxy resin. Since corrosions in the actual fields simultaneously affect the lap-spliced rebars and stirrups, our testing program considered both of these corrosions.

In this study, we fabricated 12 RC beams exhibiting sufficient and insufficient lap-splice lengths. The beams were corroded using electrochemically accelerated corrosion. Thereafter, the electrochemically corroded beams were subjected to a four-point bending test. The behaviors of these corroded beams were compared with those of similar non-corroded beams tested in our previous study [54].

## 2. Materials and Methods

### 2.1. Test Specimens

#### 2.1.1. Specimen Fabrication and Configuration

**Figure 1:**
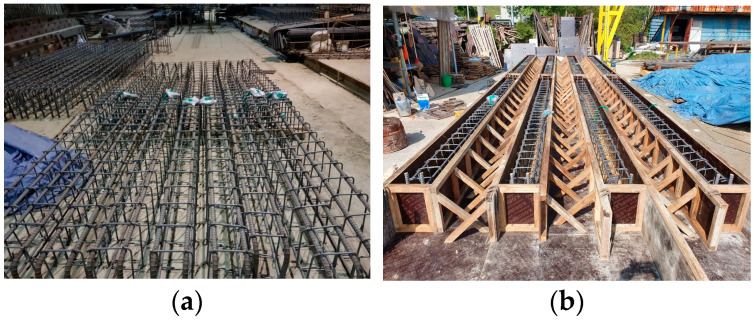
Beam fabrication setup: (**a**) reinforcement cage assembled for the beam specimens and (**b**) wooden mold with reinforcement cage prior to casting.

Concrete beam specimens were prepared using Ordinary Portland Cement (Type I) with a water-cement ratio of 0.40–0.50 and 10% fly ash (by mass of cement). Coarse aggregate with a maximum size of 25 mm was used, and the slump was maintained at up to 150 mm to ensure adequate workability. All concrete materials, including cement, aggregates, and fly ash, were sourced by, and the concrete was manufactured by Hankuk Remicon Co., Ltd., Hwaseong-si, Gyeonggi-do, Republic of Korea. Reinforcement cages were assembled prior to casting (Figure 1a) and positioned in wooden molds (Figure 1b). Concrete was poured into the molds and compacted using vibration to remove entrapped air. The beams were demolded after 24 hours and cured in water for 28 days.

**Figure 2:**
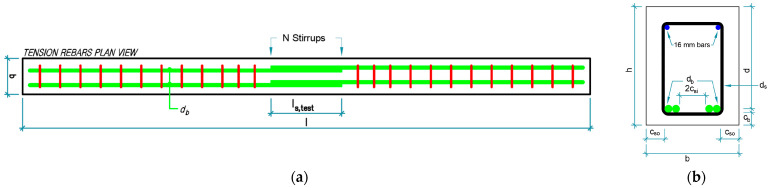
Specimen configurations: (**a**) longitudinal plan and (**b**) transverse section views [54].

Twelve RC beams were cast, exhibiting the following dimensions: width (b), 310 mm; height (h), 395 mm; and overall length (l), 4900 mm. The diameters of the longitudinal tensile rebars, $d_{b}$, was 25 mm and that of compression rebars was 16 mm. The longitudinal tensile rebars featured a lap-splice length, $l_{s,test}$, of 610 mm (24$d_{b}$), positioned at the center of the beam. The specimens were classified into four groups of three beams each (Table 1) and denoted using the notation, “CaaYbbScc,” where “aa” is the 28-day compressive strength of the beams, $f_{c}^{\prime }$, measured by testing concrete cylinders; “bb” is the yield strength of longitudinal rebars and stirrups, $f_{y}$; and “cc” is the diameter of the stirrups, $d_{s}$, in the lap-splice region. Further, N is the number of stirrups in the lap-splice region; d is the effective depth of the beam; $c_{b}$ and $c_{so}$ are the bottom and side covers of longitudinal rebars, respectively; and $c_{si}$ is half of the clear spacing of lap-spliced rebars. These parameters are graphically shown in Figure 2.

**Figure 3:**
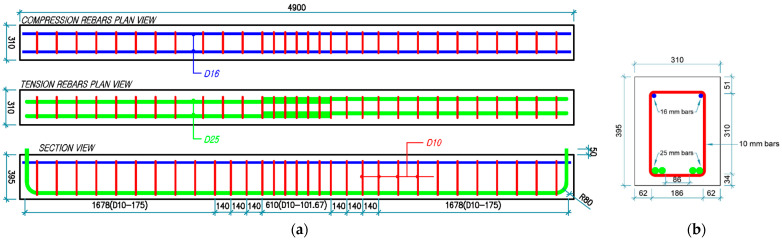
Reinforcement details for Group 1 (C27Y500S10) and Group 3 (C27Y300S10) beams: (**a**) longitudinal and (**b**) transverse views [54].

**Figure 4:**
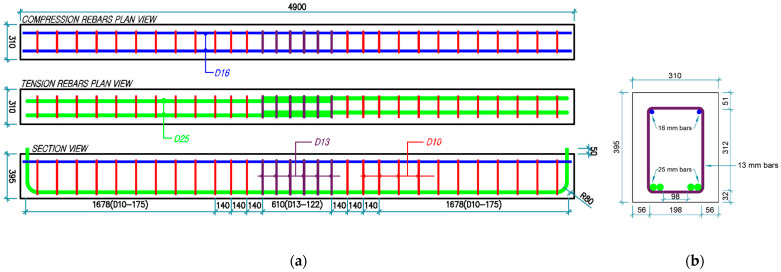
Reinforcement details for Group 2 (C27Y500S13) and Group 4 (C24Y300S13) beams: (**a**) longitudinal and (**b**) transverse views [54].

Figure 3 and Figure 4 detail the rebar configurations and dimensions of all four beam groups. Groups 1 and 2 consisted of longitudinal tensile rebars and stirrups with a yield strength of 500 MPa, as compared to the 300 MPa yield strength specified for Groups 3 and 4. The beams of Groups 1 and 3 utilized seven 10 mm stirrups at a center-to-center spacing of 101.67 mm in the lap-splice region, whereas those of Groups 2 and 4 utilized six 13 mm stirrups at a spacing of 122 mm. Stirrups of 10 mm diameter were used except in the lap-splice region.

#### 2.1.2. Lap-Splice Length

The American Concrete Institute Building Code (ACI 318-19) [55] recommended the following equation for calculating the lap-splice length, $l_{s,ACI}$:

(1) $$l_{s,ACI}=\left(\frac{3}{40}\frac{f_{y}}{\lambda \sqrt{f_{c}^{\prime }}}\frac{\Psi_{t}\Psi_{e}\Psi_{s}\Psi_{g}}{\left(\frac{c_{b}+K_{tr}}{d_{b}}\right)}\right)d_{b}$$

where

(2) $$K_{tr}=\frac{40A_{tr}}{sn}$$

where $A_{tr}$ is the area of the transverse reinforcement crossing the potential splitting plane (in^2^); $c_{b}$ is the minimum concrete cover (distance between the center of the rebar to the closest surface) or one-half of the center-to-center spacing of the rebars (in); $d_{b}$ is the diameter of the rebar (in); $f_{y}$ and $f_{c}^{\prime }$ are the yield and compressive strengths of the rebar and concrete, respectively (psi); n is the number of spliced rebars; s is stirrup spacing (in); $\psi_{t}$ is the casting-position factor (its value is 1.3 if more than 12 in of concrete is placed below the horizontal reinforcement and 1.0 for other cases); $\psi_{e}$ is the coating factor (its value is 1 for non-coated rebar and 1.3 or 1.5 for epoxy coating, depending on the cover and spacing conditions); $\psi_{s}$ is the rebar size (diameter) factor (its value is 1 for rebars with 22 mm or higher diameter rebars and 0.8 for smaller-diameter rebars; $\psi_{g}$ is the reinforcement grade (yield strength) factor (its value is 1 for a 40 or 60 ksi rebar, 1.15 for an 80 ksi rebar and 1.3 for a 100 ksi rebar, i.e., 1 and 1.15 for 300 and 500 MPa rebars, respectively); λ is the lightweight factor (its values are 1 and 0.75 for normal-weight and lightweight concretes, respectively); and the confinement term (($c_{b}+K_{tr})/d_{b}$) shall not be more than 2.5.

Lap-splice lengths as per ACI 318-19, $l_{s,ACI}$, as well as tested lap-splice length, $l_{s,test}$, for all four beam groups are presented in Table 1. Groups 1 and 2 were designed to exhibit bond failure owing to insufficient lap-splice length, i.e., $l_{s,test}\;$ < ${\;l}_{s,ACI}$, whereas Groups 3 and 4 were designed to fail by yielding of rebars by providing a sufficient lap-splice length, i.e., $l_{s,test}$ > $l_{s,ACI}$.

#### 2.1.3. Location of the Strain Gauges

**Figure 5:**
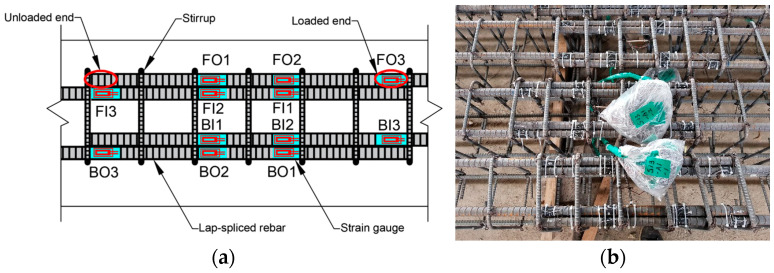
Location of the strain gauges for Group 1 (C27Y500S10) and Group 3 (C27Y300S10) beams: (**a**) schematic of the gauge locations [54] and (**b**) actual gauge location.

Twelve steel strain gauges, manufactured by Tokyo Measuring Instruments Laboratory Co., Ltd. (TML), Tokyo, Japan, were installed on the lap-splice rebars in each beam to monitor the strain profile (Figure 5a). The strain gauges were protected from moisture and damage by butyl-rubber SB and VM tapes, manufactured by TML, Tokyo, Japan. When we started the test after the corrosion, approximately 80% of the strain gauges functioned properly, whereas the remainder were either inoperative or produced unreliable data. On the other hand, almost all the gauges on non-corroded beams, prepared in the same batch, were fully functional; this indicates that most of the gauges may have been damaged by corrosion. These damages could be attributed to corrosion-related sources, such as chloride solution ingress into the butyl rubber tape, leading to detachment or corrosion of the gauges, or corrosion of the gauge wires due to prolonged exposure to the chloride solution. Therefore, more robust and corrosion-resistant methods are needed to ensure gauge reliability, especially as higher corrosion levels could damage more gauges. The positions of the strain gauges are represented by a three-letter notation in Figure 5b. The first letter represents the lap-splice at the front (F) or back (B) of the beam, the second letter stands for the rebar of the lap-splice located on the outer (O) or inner (I) side, and the number (1–3) denotes the location of each strain gauge (3 is the loaded end of the lap-splice, 1 is closer to the unloaded end, and 2 is between them). Figure 5b shows the loaded and unloaded ends for one of the lap-spliced rebars, which are similar for other rebars in the setup.

#### 2.1.4. Rebar Ribs

**Figure 6:**
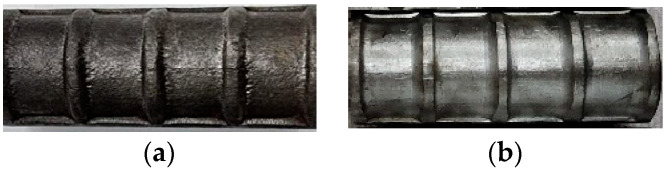
Deformation patterns of the longitudinal rebars: (**a**) TWPS and (**b**) KSHS rebars.

The deformation patterns of the lap-spliced rebars are shown in Figure 6a,b. Lap-spliced rebars made by Power Steel Co., Ltd., Kaohsiung, Taiwan (marked as TWPS), used for C27Y300S10 and C27Y500S13, had a relative rib area, $R_{r}$, of 0.06, whereas those made by Hyundai Steel, Seongnam-si, Gyeonggi-do, Republic of Korea (marked as KSHS), used for C27Y500S10 and C24Y300S13, had a relative rib area of 0.09. The relative rib area is defined as the ratio of the bearing area of the rebar (product of the nominal rebar perimeter and the rib height) to its shearing area (product of the nominal rebar perimeter and the average center-to-center spacing of the ribs) [56]. Notably, $R_{r}$ will be used to estimate the bonding strength based on the effects of the geometries of deformed rebars (Section 3.6.1).

### 2.2. Electrochemical Corrosion

**Figure 7:**
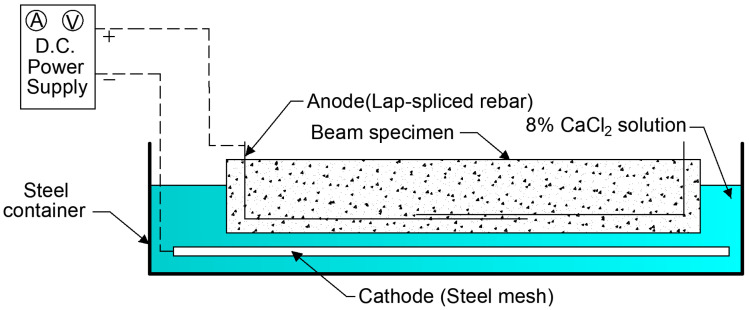
Electrochemical corrosion cell.

An electrochemical corrosion cell was developed for the accelerated corrosion of the RC beams (Figure 7). For the electrochemical corrosion, the 12 beams were placed in steel containers and connected to an electric power source (voltage, 10–12 V), TDP-305A, manufactured by Toyotech Co., Ltd., Incheon, Republic of Korea. In the cell, the lap-spliced rebars acted as the anode, and a steel mesh, which was placed below the beams, functioned as the cathode. During the fabrication, a 50 mm length of the lap-spliced rebars was deliberately left protruding from the top surface of the beams to facilitate the electrical connection to the anode. As both rebars in each lap-splice were firmly connected, only one rebar per lap-splice (two rebars per beam) was connected to the direct current supply for accelerated corrosion. Additionally, an 8% CaCl_2_ aqueous solution was utilized as an electrolyte to complete the circuit.

A weekly wetting-drying cycle was established to achieve corrosion results similar to the natural corrosion process. This cyclic exposure facilitated chloride ingress and oxygen availability during the wetting and drying periods, respectively, thereby effectively accelerating corrosion. Almost half the beam depth was submerged in the aqueous solution during the wetting period (four days), after which water was removed using a pump during the drying period (three days). To accelerate the transfer of the aqueous solution through the concrete cover to the lap-splice rebars, a few holes (3–5) with a diameter of approximately 4 mm were drilled into the side of the beams in the vicinity of longitudinal tensile rebars within the lap-splice region.

### 2.3. Test Setup

**Figure 8:**
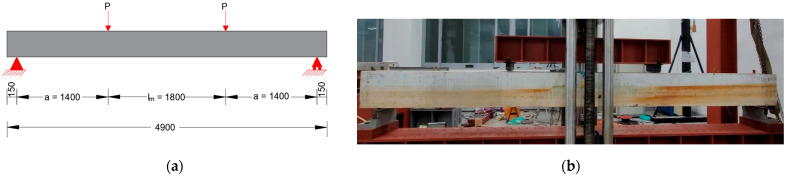
Test setup: (**a**) schematic of the test setup and (**b**) actual test setup [54].

The beams were tested, approximately 22 months after fabrication, under displacement-controlled four-point bending at the rate of 3.47 mm/min using a Universal Testing Machine (UTM) of 200 tf capacity, from M & T Korea Co., Seoul, Republic of Korea. The beams were tested such that they had a simply supported span of 4600 mm and a constant moment region, $l_{m}$, of 1800 mm (Figure 8a). Steel pedestals were positioned as supports at 150 mm from the beam ends, and the load was applied through a steel spreader beam (Figure 8b). Additionally, a load cell was placed in the middle of the spreader beam to measure the load, and two displacement transducers were installed at the midspan to measure the deflection of the beam. Sensor measurements were recorded with a Portable Data Logger, TDS-303, manufactured by TML, Tokyo, Japan. Unfortunately, a computer program crash during an experiment meant that no data were retrieved for the beam CC27Y500S10-3, except for the peak load.

### 2.4. Corrosion Measurement

Following the beam tests, demolition hammers were used to break and remove the concrete, allowing the rebars to be extracted for corrosion measurements. Three rebar samples were cut from each lap-splice rebar, and three to four were cut from the stirrups within the lap-splice region for each beam. The rebar samples were deliberately taken from locations other than the strain gauge placements, as their surfaces were locally ground at these locations to facilitate strain gauge attachment. Bars were cut from rib to rib to ensure the precise measurement of each sample. All rebar samples were cleansed using Clarke’s solution, following ASTM G1-03 [57]. Thereafter, they were weighed to measure the mass loss against non-corroded rebars. The mass loss due to corrosion, C, is referred to as the “corrosion level” and is given by the following equation:

(3) $$C=\frac{w_{n}-w_{c}}{w_{n}}\times 100$$

where $w_{n}$ and $w_{c}$ are the masses per unit length of the non-corroded and corroded rebars, respectively.

## 3. Results and Analysis

### 3.1. Failure Mode

**Figure 9:**
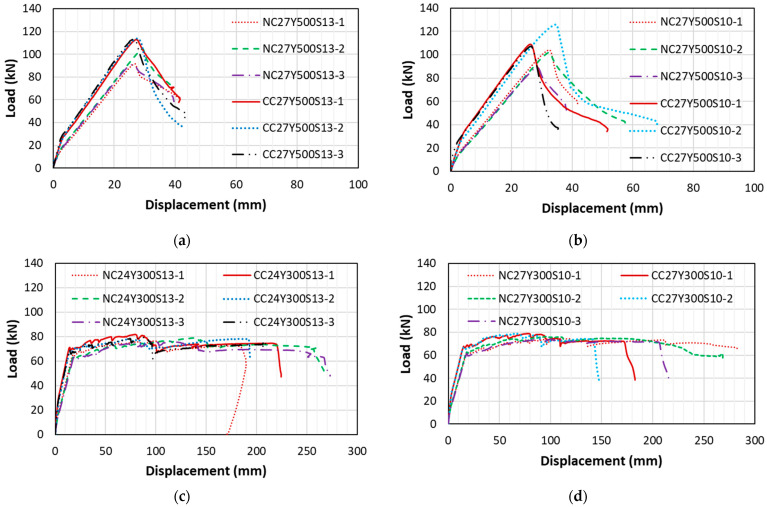
Load–displacement curves of the (**a**) C27Y500S10, (**b**) C27Y500S13, (**c**) C27Y300S10, and (**d**) C24Y300S13 beams.

Figure 9 shows the load–displacement curves of the corroded beams (tested in this study) and the non-corroded beams (tested in our previous study) [54]. Letter C (for corroded beams) or N (for non-corroded beams) was added at the beginning of the beam notations to differentiate them. To further distinguish between replicates, a hyphenated number (1–3) was appended to the end of the group notation to identify the three beams in each group. To verify the concrete strength during the test, control concrete cylinders, which were stored in the same environment as the corresponding beam specimens, were tested concurrently to measure their compressive strengths. Groups 1–3 had a 28-day compressive strength, $f_{c}^{\prime }$, of 27 MPa, whereas Group 4 had $f_{c}^{\prime }$ of 24 MPa (Table 1). However, at the time of testing, the measured compressive strengths were found to be higher than $f_{c}^{\prime }$. Specifically, Groups 1–4 exhibited compressive strengths of 33.2 MPa (22.5% higher than $f_{c}^{\prime }$), 28.5 MPa (3.6% higher), 27.5 MPa (2.8% higher), and 26.3 MPa (12.2% higher), respectively. Similar results were reported by Tastani and Pantazopoulou, where corroded beams showed a 25% increase in compressive strength of concrete [58]. To compare the bonding behaviors of the corroded and non-corroded beams (Section 3.6), the stresses in the corroded lap-spliced rebars were calculated using 28-day compressive strength, i.e., $f_{c}^{\prime }$. For the C27Y500S10 and C27Y500S13 beams, the provided lap-splice length of 610 mm was less than that recommended by ACI 318-19, $l_{s,ACI}$ [55]. That is why the bond failure of lap-spliced rebars happened before the yielding of rebars, thereby exhibiting a brittle failure. Load–displacement curves of these beams (Figure 9a,b) display an increase in the curve until the peak load. Thereafter, it decreased rapidly until failure. This behavior represents a brittle failure due to the sudden loss of bond between the rebar and the surrounding concrete in the lap-splice region.

For the C27Y300S10 and C24Y300S13 beams, the provided lap-splice length met the requirements of ACI 318-19 [55]. These beams exhibited sufficient bond strength and failed by the yielding of rebars. The load–displacement curves (Figure 9c,d) increased linearly up to the initial cracking of the beams in the tension region. Afterward, the slope reduced slightly, continuing up to the yielding point. Beyond yielding, the beams exhibited significant ductile displacements with a minimal load increase, followed by a substantial load reduction toward the end of the curve, indicating a ductile mode of failure.

### 3.2. Corrosion Level and Load-Carrying Capacity

**Figure 10:**
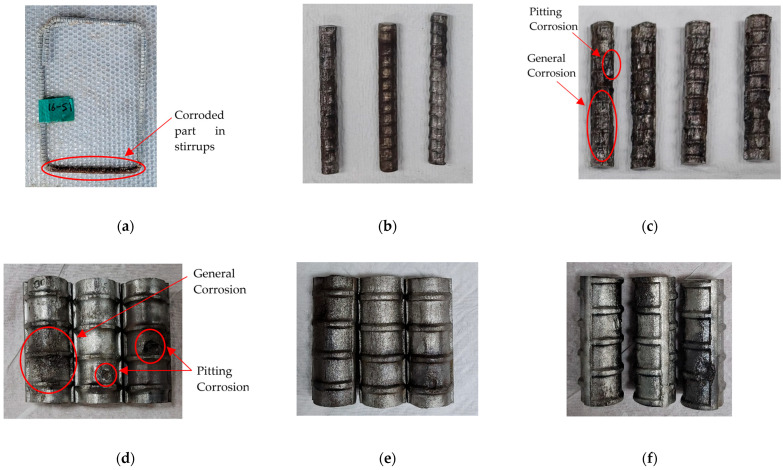
Rebars extracted for corrosion measurements: (**a**) whole stirrup (CC27Y500S13-1); (**b**) parts of the 10 mm stirrups (CC27Y300S10-1); (**c**) parts of the 13 mm stirrups (CC27Y500S13-1); (**d**) parts of the lap-spliced rebars from beams with insufficient lap-splice length (CC27Y500S10-2); (**e**) parts of lap-spliced rebars from beams with sufficient lap-splice length (CC24Y300S10-1) and (**f**) parts of the lap-spliced rebars from beams with sufficient lap-splice length (CC24Y300S13-1).

Figure 10 shows pictures of the extracted rebars for corrosion measurement. Figure 10a shows that corrosion predominantly affected the lower areas of the stirrups. Therefore, samples for 10 and 13 mm stirrups were deliberately cut from these severely corroded regions for mass-loss measurements, as shown in Figure 10b,c, respectively. Pictures of the extracted lap-spliced rebars for corrosion measurement are shown in Figure 10d–f. The rebars exhibited both general and pitting corrosion, as indicated in Figure 10c,d. General corrosion uniformly affects a large area of the rebar, whereas pitting corrosion is a localized corrosion phenomenon that leads to the formation of small holes or pits in the steel. The observed pits on lap-spliced rebars consisted of small pits of approximately 2 mm width and 1 mm depth, and large pits of up to 5 mm width and 2 mm depth.

Table 2 summarizes the average corrosion levels for the stirrups, $C_{t}$ and lap-splice rebars, $C_{l}$, along with the peak or maximum loads for the corroded and non-corroded beams, $P_{C}$ and $P_{N}$, respectively. Stirrup corrosion levels ranged from 4% to 18%. For the lap-splice rebars, the KSHS rebars showed higher corrosion (up to 3.17%) compared to the TWPS rebars (around 1%). It is observed that corroded beams exhibited higher load-carrying capacities than their non-corroded counterparts. Despite exhibiting significant corrosion (up to 18% mass loss), stirrups degradation did not cause any reduction in the load-carrying capacity of the beams. This finding indicated that the observed level of stirrup corrosion was not sufficiently severe to cause the loss of concrete confinement. The percentage increase in the load-carrying capacity of the corroded beams with respect to (w.r.t.) the average load-carrying capacity of the non-corroded beams of each group, $P_{N,avg}$, is also presented in the last column of Table 2. The corroded beams with bond failure exhibited a higher increase in the peak load (28% w.r.t. the non-corroded beams) than the corroded beams (6% w.r.t. the non-corroded beams) with yielding failure. This indicated that the low corrosion level significantly contributed to the increase in the bond capacity of the lap-splices, which consequently increased the load-carrying capacity of the beams, as explained below.

Low corrosion levels initially increased bond strength via two mechanisms: increased rebar surface roughness, which enhanced frictional stress between the rebar and surrounding concrete, and the formulation of the expansive rust particles on the surface of the rebars, which increased the concrete–rebar mechanical interlock [16,17,18,19,20]. These combined effects resulted in a significant increase in the peak loads of all corroded beams w.r.t. those of non-corroded beams. However, higher corrosion levels can result in the reduction in adhesion between rebar and concrete, causing a reduction in bond performance. As presented in Table 2, no direct correlation is seen between the increased load-carrying capacity and corrosion level. For instance, CC27Y500S10-1 and CC27Y500S10-2 exhibited similar corrosion levels but significantly different load-carrying capacities. The observed disparity in the load-carrying capacities of corroded beams can be attributed to the corrosion type and location. General and pitting corrosion occurred along the lap-spliced rebars. For low corrosion levels, general corrosion tended to promote the increase in the bond strength of the lap-splices, as already discussed above. Conversely, pitting corrosion at a critical location, such as the lap-spliced rebar ribs at the loaded end, tended to decrease its bond strength. Since it is hard to control the location of the pitting corrosion, there are uncertain corrosion effects of lap-splice on its bonding strength especially for low corrosion levels. The adverse effects of corrosion might be apparent for high-level corrosion because pitting corrosion would most likely occur at the critical location.

### 3.3. Cracking Patterns According to the Failure Mode

No significant corrosion-induced cracks were observed in the corroded beams; only corrosion stains were visible, primarily on the bottom surface. This localization occurred because the electrochemical corrosion was targeted at the lower part of the beams, where the bottom concrete cover for the tensile rebars was less than the side cover for the tensile rebars. Critically, the corroded beams exhibited the same crack pattern as the non-corroded beams, as will be discussed below.

**Figure 11:**
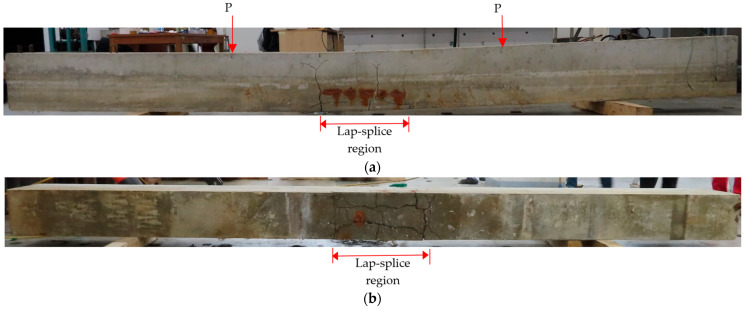
Crack pattern of the beam with insufficient lap-splice lengths (CC27Y500S10-1): (**a**) side and (**b**) bottom views.

Figure 11, which includes the loading points and lap-splice region, presents the side and bottom views of the CC27Y500S13-1 beam as a representative example of the typical crack pattern of beams with lap-splice bond failure. Specifically, Figure 11a shows vertical cracks initiating at the bottom side and extending nearly to the top of the beams. Some beams also exhibited longitudinal cracks at the sides in the vicinity of the tensile lap-spliced rebars (Figure 11a). Additionally, significant longitudinal cracks developed along the lap-spliced rebars, and transverse cracks appeared along the full width of the beam bottom, specifically at the ends of the lap-splice region (Figure 11b).

**Figure 12:**
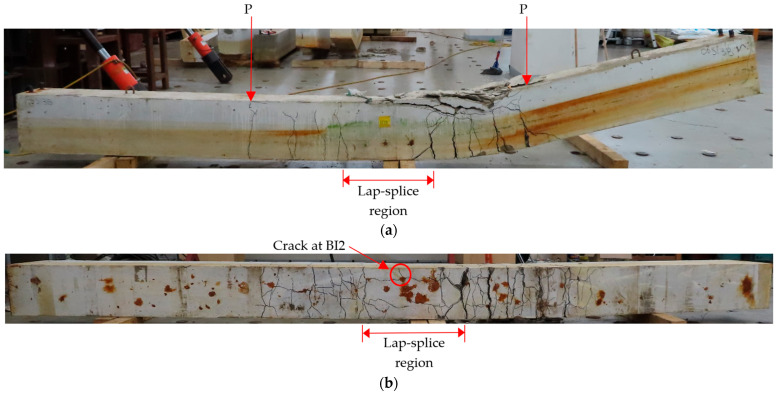
Crack patterns of the beam with sufficient lap-splice length (CC24Y300S13-2): (**a**) side and (**b**) bottom view.

Figure 12, which includes the loading points and lap-splice region, presents the side and bottom views of the CC24Y300S13-2 beam as a representative example of the typical crack pattern of beams which failed after rebar yielding. At first, transverse flexural cracks were initiated at the ends of the lap-splices just before the yielding of beams. After that, several other transverse flexural cracks appeared away from the lap-splice toward both loading point locations, starting from the bottom of the tension region, growing toward the compression region. At the bottom of the beam, small longitudinal cracks appeared in the lap-splice region, and significant transverse cracks occurred at the end of the lap-splice region and towards the both loading point locations. After the peak load, the concrete crushed in the compression region. A crack at Gauge BI2 is highlighted in Figure 12b to elucidate the strain behavior to be discussed in the next section.

Beams with insufficient lap-splice lengths failed in a brittle manner at small midspan displacements, exhibiting small cracks in the lap-splice region. However, beams with sufficient lap-splice lengths failed in a ductile manner at larger midspan displacements, exhibiting significant cracks both within and outside the lap-splice region. This failure pattern indicated that an insufficient tensile lap-splice length precipitates sudden failure without significant prior visual warning via concrete cracking.

### 3.4. Strain in Lap-Spliced Rebars

**Figure 13:**
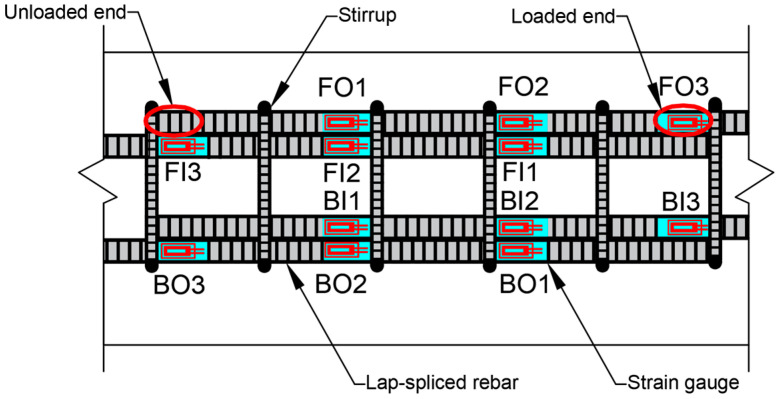
Locations of the strain gauges for beams C27Y500S13 and C24Y300S13 [54].

Figure 13 shows the location of the strain gauges for beams C27Y500S10 and C24Y300S10 (the locations of the strain gauges for beams C27Y500S13 and C24Y300S13 are already shown in Figure 5b). The strain–displacement curves of the corroded and non-corroded beams were generally identical.

#### 3.4.1. Beams with Insufficient Lap-Splice Lengths

**Figure 14:**
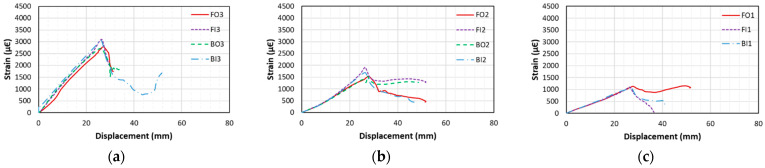
Strain–displacement curves of the beam with insufficient lap-splice lengths (CC27Y500S10-1) for various strain gauges: (**a**) FO3, FI3, BO3, and BI3; (**b**) FO2, FI2, BO2, and BI2; and (**c**) FO1, FI2, and BI1.

Figure 14 shows typical strain–displacement curves of bond failure beams with insufficient lap-splice lengths (CC27Y500S10 and CC27Y500S13). Figure 14a–c show the strain behaviors at the following locations: the loaded end of the lap-splice, the strain gauge close to the unloaded end, and the middle of the former two, respectively. It can be observed that throughout the experiment, the strain at the loaded end was highest and decreased toward the unloaded end. For the bond failure beams, the strain increased linearly with the displacement until the peak load was reached (which occurred at the peak strain). Thereafter, this strain decreased because of bond loss. Additionally, several strain gauges, such as BO1 for CC27Y500S10-1, were damaged during the corrosion; hence, their data are not shown in the graph.

**Figure 15:**
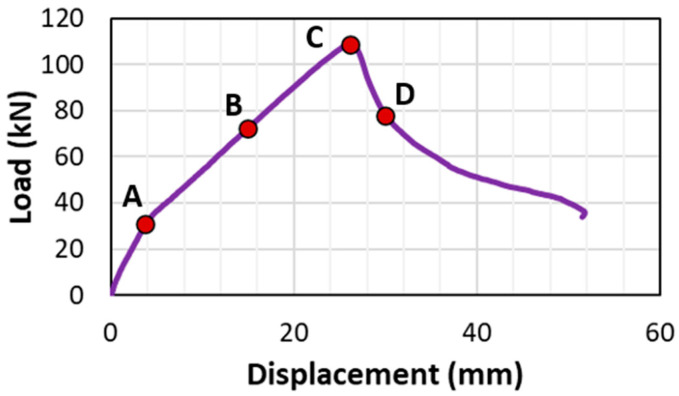
Loading stages of the beam with insufficient lap-splice lengths (CC27Y500S10−1).

Figure 15 shows the typical load–displacement curves of beams with insufficient lap-splice lengths, along with four different loading stages, A–D. Stage A represents the transition of the beam from uncracked elastic to cracked elastic. Correspondingly, a slight decrease in the slope of the load–displacement curve is shown in Figure 15, indicating the onset of tensile cracking in the beams at Stage A. Stage C represents the peak load point. Stage B represents a stage between A and C. Stage D is defined as the range along the descending branch of the load–displacement curve that follows the peak load.

**Figure 16:**
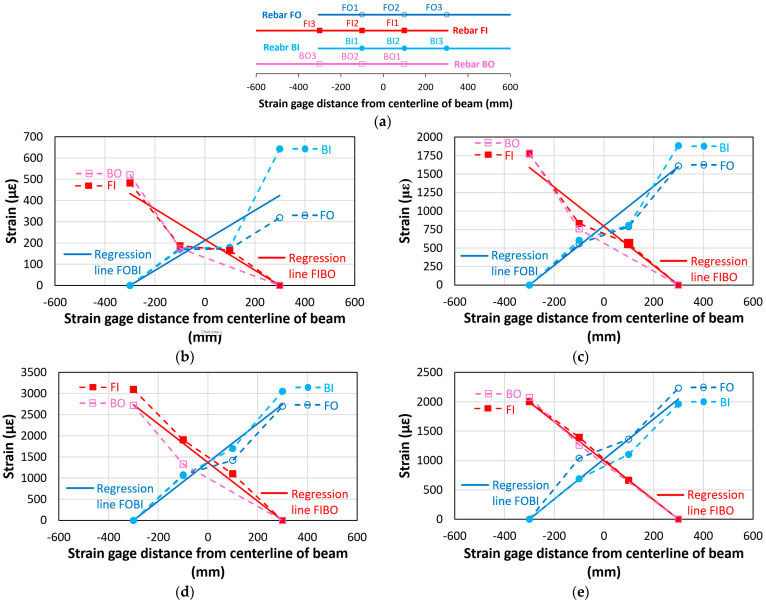
Strain values along the lap-spliced rebars for the beam with insufficient lap-splice lengths (CC27Y500S10-1) at various loading stages: (**a**) Strain gauge locations on lap-spliced rebars; (**b**) Stage A (4 mm displacement); (**c**) Stage B (15 mm displacement); (**d**) Stage C (26 mm displacement); and (**e**) Stage D (30 mm displacement).

Figure 16a–d show the strain values of the lap-spliced rebars along their longitudinal locations during the key loading stages (Stages A–D) for the bond failure beam (CC27Y500S10-1). Across all stages, the strain profile exhibited almost a linear increase from the unloaded to loaded ends of the lap-spliced rebars. Two linear regression lines for rebars having symmetrical strain distribution are also indicated in Figure 16, i.e., “Regression line FOBI” for rebars FO and BI and “Regression line FIBO” for rebars FI and BO. Zero strain was assumed at the unloaded end of the lap-splice. Strain gauge BO1 was found not to be working during experiment; therefore, no strain values are shown for that strain gauge. Figure 16a shows that at the initial loading stage (Stage A, 4 mm displacement), very low strain was developed in the lap-spliced rebars. Figure 16b shows the strain values at Stage B (15 mm displacement), representing the gradual increase in the rebar strain with increasing displacement. Figure 16c shows the highest recorded strain in the lap-spliced rebars at the maximum loading (Stage C, 26 mm displacement). Following the peak load, a subsequent decrease in the strain values was observed at Stage D (30 mm displacement) in Figure 16d, which is directly attributable to the bond slip of lap-spliced rebars. Across all loading stages, the maximum strain consistently occurred at the loaded end of the lap-splice and decreased almost linearly toward the unloaded end.

#### 3.4.2. Beams with Sufficient Lap-Splice Lengths

**Figure 17:**
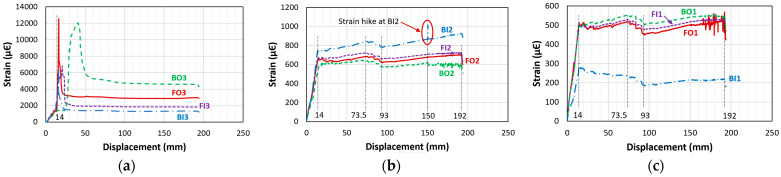
Strain–displacement curves of the beam with sufficient lap-splice lengths (CC24Y300S13-2) at (**a**) the loaded end (FO3, FI3, BO3, and BI3); (**b**) one-third of the lap-splice length from the loaded end (FO2, FI2, BO2, and BI2) and (**c**) two-third of the lap-splice length from the loaded end (FO1, FI1, BO1, and BI1).

Figure 17 shows typical strain–displacement curves of ductile failure beams with sufficient lap-splice lengths (CC27Y300S10 and CC27Y300S13). For these beams, the strain increased linearly up to a displacement of approximately 14 mm for all strain gauges. Afterward, the strain values increased suddenly (strain hike) at the loaded ends (FO3, FI3, BI3, and BO3) of the lap-splices (Figure 17a) owing to the appearance of significant transverse cracks at the loaded ends (which are the same as the ends of the lap-splices or the ends of the lap-splice region), as shown in Figure 12. The sudden increase in the BO3 strain was slightly delayed because of the unsymmetrical crack behavior (Figure 12b). The crack widths were unsymmetrical at the end of the lap-splice region in this picture. The strain values recorded at these strain hike points were significantly higher than the yield strain. Following the strain hike, the strain at the loaded ends decreased dramatically, remaining constant until the final failure of the beam (Figure 17a). Contrary to the loaded ends, the strain gauges at other locations (Figure 17b,c) exhibited a small, continuous strain increase with increasing load, until the peak load was reached at 73.5 mm displacement. Afterward, the strain decreased until the compressive concrete started crushing at a 93 mm displacement (Figure 17b,c). This decreased strain was due to the reduction in the load with the increasing displacement. After the initial crushing of the concrete in the compression region (at a 93 mm displacement), the stresses were redistributed in the beam section, and the strain in the tensile rebars increased continuously with the displacement until a 192 mm displacement (Figure 17b,c). A sudden strain hike in the strain–displacement curve of BI2 is indicated in Figure 17b, which can be attributed to the crack occurring at that location, which is marked by a red circle in Figure 12b. As the concrete crushing progressed, the beam failed eventually, and the strain values decreased after a displacement of 192 mm (Figure 17b,c).

**Figure 18:**
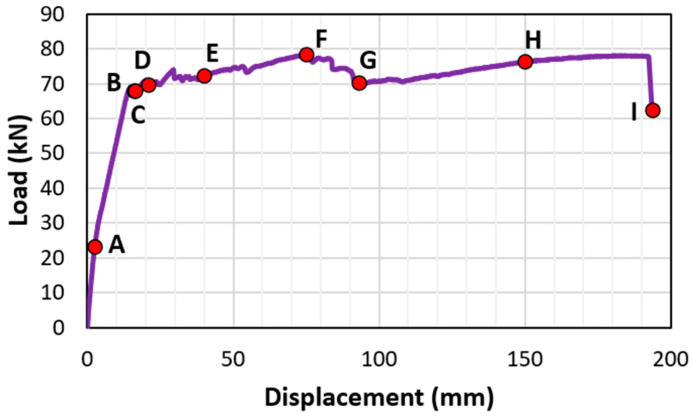
Loading stages of the beam with sufficient lap-splice lengths (CC24Y300S13-2).

Figure 18 shows the typical load–displacement curves for beams with sufficient lap-splice lengths, defining the various loading stages (Stages A–I). Stage A represents the transition from the uncracked elastic state to the cracked elastic state. Stages B, C, D, and E correspond to the specific strain hikes observed for strain gauges BI3, FO3, FI3, and BO3, respectively (Figure 17a). Stage F represents the peak load, and G corresponds to the crushing of concrete in the compression region. Stage H highlights the strain hike at the strain gauge, BO2, shown in Figure 17b, due to cracks occurring at this location, indicated in Figure 12b. Stage I represents beam failure at the end of the load–displacement curve.

**Figure 19:**
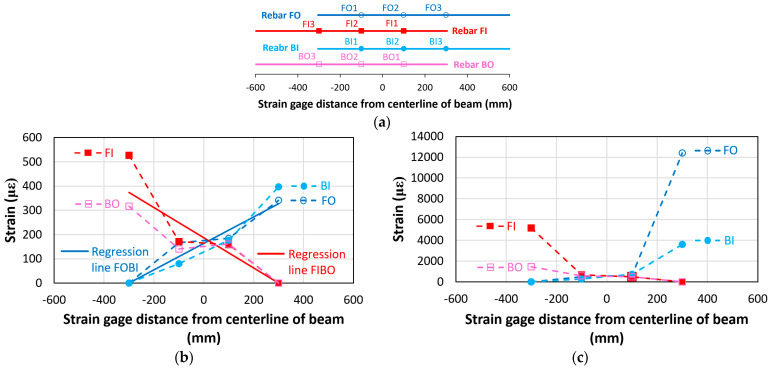

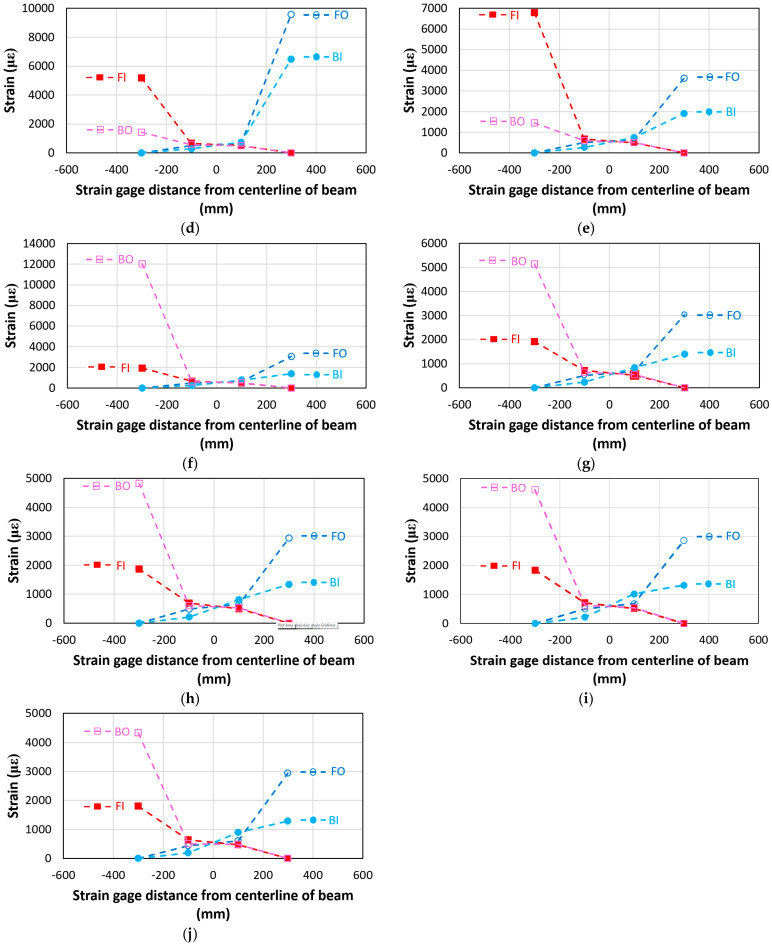
Strain values along the lap-spliced rebars for the beam with sufficient lap-splice lengths (CC24Y300S13-2) at various midspan displacements: (**a**) Strain gauge locations on lap-spliced rebars; (**b**) Stage A (4 mm displacement); (**c**) Stage B (16.2 mm displacement, BI3 peak strain); (**d**) Stage C (16.5 mm displacement, FO3 peak strain); (**e**) Stage D (21 mm displacement, FI3 peak strain), (**f**) Stage E (40 mm displacement, BO3 peak strain); (**g**) Stage F (73.5 mm displacement, peak load); (**h**) Stage G (90 mm displacement); (**i**) Stage H (150 mm displacement) and (**j**) Stage I (195 mm displacement).

Figure 19 shows the strain values of the lap-spliced rebars along their longitudinal locations at different loadings (Stages A–I) of beam CC24Y300S13-2 with sufficient lap-splice length. Similar to beams with insufficient lap-splice lengths, Figure 19 shows that maximum strain occurred at the loaded end of the lap-splices and decreases toward the unloaded end for all loading stages. Figure 19a shows the strain distribution at a displacement of approximately 4 mm, where the initial crack of the tensile region of the beams occurred (Stage A, Figure 18). Before rebar yielding, the strain linearly increased from the unloaded to the loaded ends. Figure 19a shows two linear regression lines for the rebars exhibiting a symmetrical strain distribution, i.e., “Regression line FOBI” for rebars FO and BI, and “Regression line FIBO” for rebars FI and BO. Figure 19b–e show the strain distributions corresponding to the displacements (16.2, 16.5, 21, and 40 mm) where strain hikes occurred for strain gauges BI3, FO3, FI3, and BO3 (Stages B–E, Figure 18), respectively. These strain hikes in strain–displacement curves occur at the loaded ends of rebars due to significant transverse cracks occurring at the ends of the lap-splice region (at the loaded ends of rebars), as shown in Figure 12a,b. The strain values at these strain hikes were significantly higher than the yield strain. Owing to the high localized strain at the loaded ends, the strain distribution along the lap-splice rebars was no longer linear from the unloaded end to the loaded end (Figure 19b–e). Following the strain hike, the strain at the loaded ends decreased to certain values, after which the strain across all strain gauges remained relatively constant throughout Stages F–I (Figure 19f–i). Furthermore, the strain values were not symmetric around the midspan at the both ends of lap-splices (Figure 19b–i). This asymmetry can be attributed to the non-symmetrical cracking at the ends of the lap-splice region (Figure 12).

### 3.5. Initial Stiffness and Ductility

**Figure 20:**
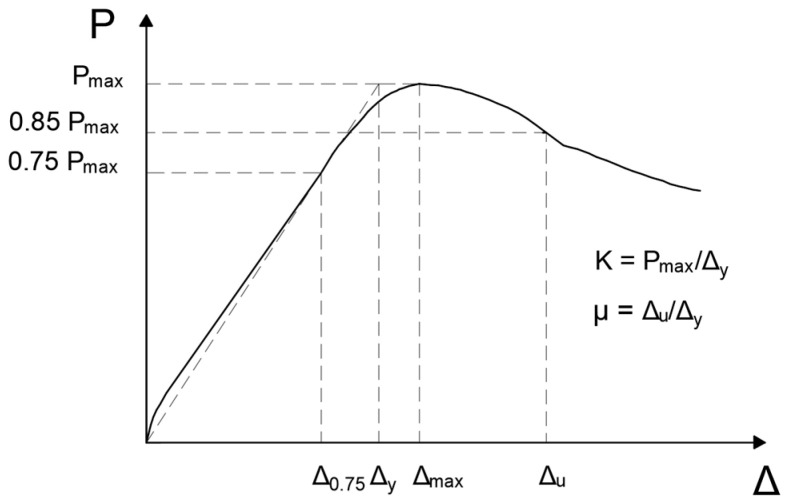
Stiffness and ductility definitions.

Figure 20 shows the typical load–displacement curve of a beam and defines the key variables necessary for calculating initial stiffness and ductility. The initial stiffness, K, of the beams is given by the following equation:

(4) $$K=\frac{P_{max}}{\Delta_{y}}$$

where $P_{max}$ is the peak (maximum) load carried by the beam, and $\Delta_{y}$ is the first yield displacement of the beam. Additionally, $\Delta_{y}$ is given by the intersection point of a straight line from the origin and passing through a point where the load reaches 75% of $P_{max}$ and a horizontal line passing through $P_{max}$ on the load–displacement curve (Figure 20) [59]. Furthermore, $\Delta_{0.75}$ and $\Delta_{max}$ are the displacements corresponding to 0.75$P_{max}$ and $P_{max}$, respectively. Further, $\Delta_{u}$ is the ultimate displacement defined as the displacement corresponding to 85% of $P_{max}$ on the declining part of the load–displacement curve [60].

The initial stiffnesses of corroded beams, $K_{C}$, and non-corroded beams, $K_{N}$, are given in Table 3. For all the corroded beams, the initial stiffnesses of the beams are found to be significantly increased as compared to their non-corroded counterparts. This increase in stiffness was due to the increased frictional stress and enhanced rebar–concrete interlocking caused by corrosion.

There are several methods to calculate ductility, including the use of displacement, curvature and energy [61]. Furthermore, the American Association of State Highway and Transportation Officials (AASHTO) and the California Department of Transportation (CALTRAN) proposed equations for calculating the ductility of bridge piers based on moment–curvature curves for cantilever and a fixed–fixed frame configurations [62,63]. However, since a cantilever is equivalent to a three-point loaded simple beam, this approach is different from the four-point loading conditions applied to our tested beams. Therefore, we cannot utilize the aforementioned ductility equations based on moment–curvature curves. The ductility or ductility index, μ, of our tested beams is calculated from the load–displacement curves as the ratio of ultimate displacement, $\Delta_{u}$, to the first yielding displacement, $\Delta_{y}$, as follows: [64].

(5) $$\mu =\frac{\Delta_{u}}{\Delta_{y}}$$

The ductility indices of all non-corroded ($\mu_{N}$) and corroded beams ($\mu_{C}$) is given in Table 3. As observed, the ductility indices of the corroded bond failure beams were more than those of non-corroded beams, with the exception of NC27Y500S13-3, whose ductility index was significantly more than those of other corroded as well as non-corroded beams in this group. This increase in the ductility index of corroded beams was due to the increased initial stiffness (decreased $\Delta_{y}$) and the increased bond strength of the corroded beams.

The ductility indices of the beams with sufficient lap-splice lengths did not exhibit any consistent effect for low corrosion levels. CC27Y300S10-1 and CC24Y300S13-2 exhibited ductility indices similar to those of the non-corroded beams, indicating that corrosion did not affect the ductility of the beams. Furthermore, the corroded beams (CC27Y300S10-2, CC24Y300S13-1, and CC24Y300S13-3) exhibited lower ductilities than their non-corroded counterparts. According to Ahmad and Barker, a displacement ductility of 3–5 was adequate for a seismic design. However, they used a conservative definition for displacement ductility, i.e., the ratio of displacement corresponding to the maximum load ($\Delta_{max}$, Figure 20) to the displacement of the first yielding of tensile rebars [65,66]. Based on the latter definition, more conservative ductility values of 4.2, 5.3, and 5.7 were obtained for CC27Y300S10-2, CC24Y300S13-1, and CC24Y300S13-3, respectively. Hence, the residual ductilities of these beams were still sufficient to satisfy the seismic design requirement. This disparity in ductility indices of corroded beams may be due to the corrosion level and corrosion type (general and pitting corrosion), i.e., higher corrosion levels and pitting corrosion can result in lower ductility.

### 3.6. Bond Strength and Stresses in Rebars with Insufficient Lap-Splice Lengths

#### 3.6.1. Bond Strength

In this section, a comparison of the bond strengths of lap-splices measured from our beam tests (with insufficient lap-splice lengths) and those obtained from the empirical equations mentioned in our previous study [54]. The bond strength of lap-splices from the beam tests, $u^{T}$, is given by the following equation:

(6) $$u^{T}=\frac{A_{b}f_{s}}{\pi d_{b}l_{s}}$$

where $A_{b}$ and $d_{b}$ are the area and diameter of the lap-splice rebars, respectively; $l_{s}$ is the lap-splice length; $f_{s}$ is the failure stress of steel, calculated by the section analysis of beams with continuous rebars instead of lap-splices, as follows:

(7) $$f_{s}=\frac{M_{test}}{A_{s}jd}$$

(8) $$M_{test}=Pa$$

where $M_{test}$ is the maximum moment at failure; a is the distance between the loading point and support, as shown in Figure 8a; $A_{s}$ is the area of the steel rebars; and jd is the moment arm (from the center of tensile rebars to the center of concrete in compression). The results are presented in Table 4. Notably, $E_{c}$ and $E_{s}$ in the table are the elastic moduli of the concrete and rebar, respectively; n is the modular ratio; and ρ is the reinforcement ratio defined as follows:

(9) $$\rho =\frac{A_{s}}{bd}$$

where b and d are the width and effective depth of the beam, respectively. Some empirical equations for predicting the bond strength of lap-splice, u, are presented in Table 5.

Table 6 presents the tested bond strengths of corroded and non-corroded lap-splices, along with the predicted bond strengths obtained by the three empirical equations in Table 5, i.e., the equations proposed by Orangun et al. [67], Darwin et al. [68], and Esfahani and Kianoush [69]. The corroded lap-splices of group C27Y500S10, with a corrosion level of 2.34–3.17%, exhibited bond strengths of 5.04–5.93 MPa, which were 5.29–7.17% higher than the average bond strength of non-corroded lap-splices and 13.00–45.34% higher than the predicted values. Similarly, the corroded lap-splices of group C27Y500S13, with a corrosion level of 0.78–1.02%, exhibited bond strengths of 5.28–5.30 MPa, which were 14.12–15.42% higher than the average bond strength of non-corroded lap-splices and 20.27–26.54% higher than the predicted values. This enhanced bond strength was attributed to increased frictional stress and improved mechanical interlock between rebars and surrounding concrete at low corrosion levels. These results are consistent with those of previous studies, demonstrating that bond strength can increase at low corrosion levels [19,39,70]. However, at higher corrosion levels, a decrease in the bond strength of the corroded rebars is expected [40,41]. The results in Table 6 indicate that the existing empirical equations can be used to conservatively estimate the bond strength of lap-splices with no corrosion or low corrosion levels.

#### 3.6.2. Verification of the Measured Stress with Theoretical Values

**Figure 21:**
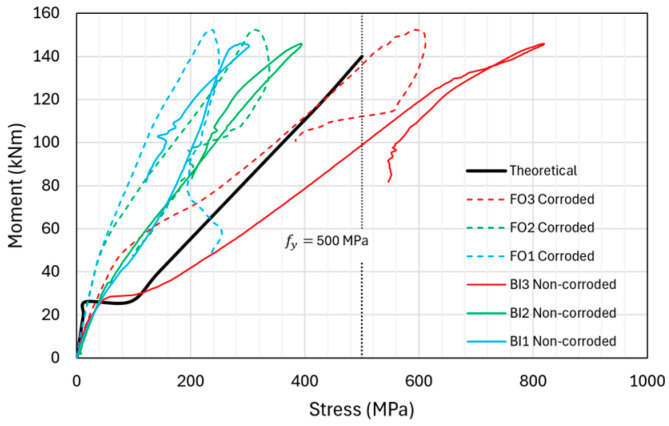
Moment–stress curves obtained from the strain-gauge data and section analysis.

Figure 21 shows the experimental moment–stress curves obtained from three strain gauges on one corroded rebar (FO1C, FO2C, and FO3C) and one non-corroded rebar (BI1N, BI2N, and BI3N), along with the theoretical moment–stress curves from the section analysis of the beam. As shown in Figure 21, the stress in the non-corroded rebar was higher than that in the corroded rebar. However, this trend did not apply to all the beams. As the corroded lap-spliced rebars had low corrosion levels, the strain gauge data for the corroded and non-corroded rebars exhibited similar results. Linear elastic section analysis (for both uncracked and cracked sections) was performed for theoretical moment–stress curves until the yield stress of 500 MPa of the tensile rebars, as indicated in Figure 21. Non-linear behavior beyond yielding was not considered because these beams showed linear elastic behavior until the peak load and failed in a brittle manner.

Here, we first assumed a linear stress–strain relationship, after which the stress, σ, in rebar is determined as follows:

(10) $$\sigma =\varepsilon \times E_{s}$$

where ε is the strain in rebar (obtained from the strain gauges), and $E_{s}$ is the elasticity modulus of rebar (220,000 MPa). For theoretical calculation, stress in tensile rebars was determined using the transformed area method. The whole section of the beam initially resisted bending; the top and bottom sides were under compression and tension, respectively. However, after the applied moment exceeded the cracking moment, the concrete in the tension part was neglected, and only the tensile rebars carried the tension. The cracking moment of the beam is given by the following equation:

(11) $$M_{cr}=\frac{f_{r}I_{g}}{y_{t}}$$

where $f_{r}$ is the rupture modulus of concrete, $I_{g}$ is the gross moment of inertia of the section, and $y_{t}$ is the distance from the centroid to the extreme tension fibers before cracking. The modulus of rupture of concrete as per ACI 318 is expressed as follows:

(12) $$f_{r}=0.62\lambda \sqrt{f_{c}^{’}}$$

where λ is the modification factor for lightweight concrete (taken as 1.0 for normal-weight concrete).

As shown in Figure 21, the measured stress of some parts of the moment–stress curves of the loaded ends, i.e., BI3 and FO3, exceeded the theoretical stress. This is because we assumed two continuous longitudinal tensile rebars without lap-splices in the section analysis of the beam. This assumption provides a uniform tensile stress distribution along the rebar length. However, the bonding behavior of lap-splices produced a non-uniform stress distribution along the lap-spliced rebars, i.e., minimum and maximum stresses at the unloaded and loaded ends of the splice, respectively. The measured stress at the loaded end significantly exceeded the theoretical stress, whereas the measured stress at other longitudinal locations was lower than the theoretical prediction.

Furthermore, the calculated tensile stress at the loaded ends, i.e., FO3C and BI3N, assuming linear behavior in lap-spliced rebars, is significantly higher than the yield stress of 500 MPa. This significant increase is not expected for actual non-linear behavior of the beam, as the rebars usually show essentially no increase in stress after yielding. There are various stress–strain models for non-linear behavior of rebar, which can be used to calculate stress after the yielding stress of rebar, e.g., elastic-perfectly plastic (constant stress after yielding) and elastic-plastic with strain hardening. The stress calculated through those models, after yield stress, would be less than that shown in Figure 21, where linear stress–strain was assumed.

#### 3.6.3. Stress Distribution in Rebars with Insufficient Lap-Splice Lengths

**Figure 22:**
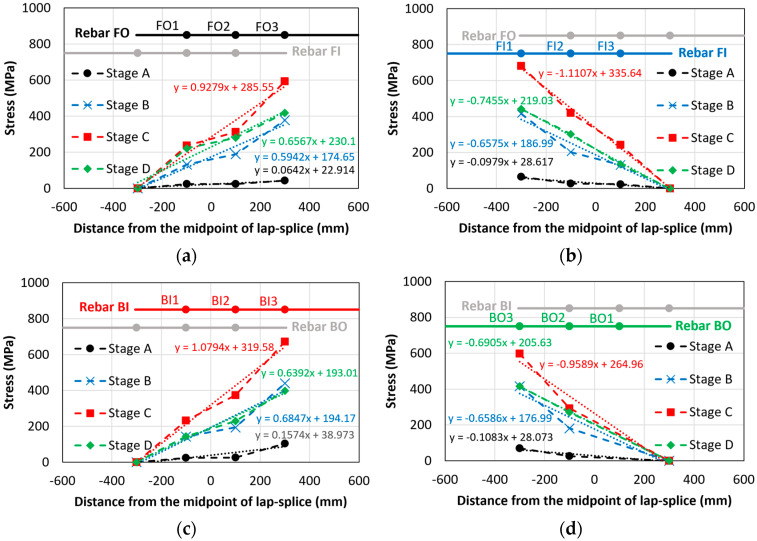
Stress distributions in rebars of corroded beams with insufficient lap-spliced lengths (CC27Y500S10-1): (**a**) rebar FO; (**b**) rebar FI; (**c**) rebar BI and (**d**) rebar BO.

**Figure 23:**
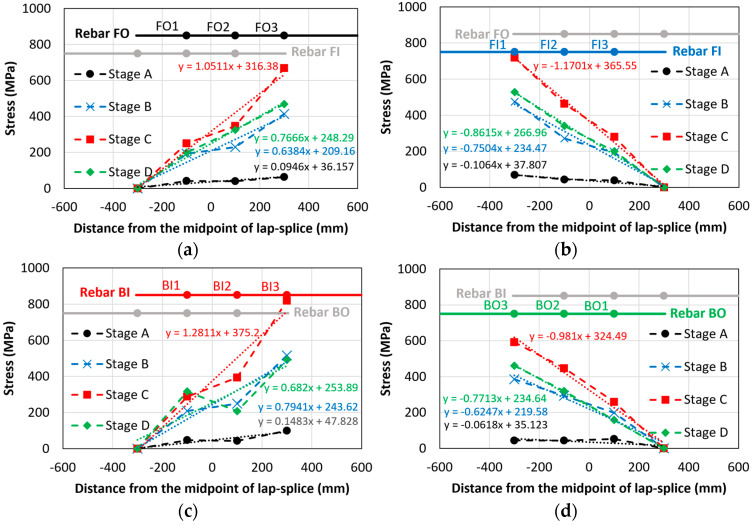
Stress distributions in rebars of non-corroded beam with insufficient lap-splice lengths (NC27Y500S10-1): (**a**) rebar FO; (**b**) rebar FI; (**c**) rebar BI and (**d**) rebar BO.

Figure 22 and Figure 23 show the stress distributions of the four tensile lap-spliced rebars under various loading conditions of corroded and non-corroded beams, respectively. The primary observation was that the stress distribution almost linearly increased from the unloaded end to the loaded end. Within these figures, thin dotted lines represent the linear regression lines (with their respective equations also shown in the same color) for each loading stage. Stages A–D for the beams with insufficient lap-splice lengths are shown in Figure 15. The slope of the regression equation increased from Stage A until the peak load (Stage C), after which it decreased owing to bond loss.

Table 7 presents a comparison of the slope values derived from the stress-distribution equations for corroded and non-corroded beams. These slopes represent the bonding behavior of lap-splices. For a given tensile force, shorter splices create steeper stress gradients owing to their higher bond-demand concentration at the loaded end, whereas longer splices exhibit more gradual stress variation. No consistent trend was observed in the stress-slopes; the slopes of corroded beams were neither uniformly higher nor lower than their non-corroded counterparts across specific loading stages. This lack of a clear relationship was attributed to the relatively low corrosion levels in this study. However, for higher corrosion levels, a consistent and pronounced trend in stress distribution of lap-splices could be observed. In both corroded and non-corroded beams with insufficient lap-splice length, the effect of wedge action is particularly evident at the loaded ends due to stress concentration. The wedge action between the rebar ribs generates radial stresses in the surrounding concrete, initiating internal cracks. These cracks propagate towards the surface, leading to splitting of the concrete cover and loss of confinement. The reduction in confinement causes rebar slip relative to the concrete, ultimately resulting in brittle bond failure. Consequently, we observed a decrease in the stress-slope values in Stage D.

## 4. Conclusions

In order to evaluate the flexural performance of old RC bridge piers with lap-splices, we fabricated 12 small-scale RC beams (four groups of three beams each) with sufficient and insufficient lap-splice lengths. Next, we electrochemically corroded the beams, after which we subjected them to a four-point bending test. The 12 beams exhibited two primary failure modes: six specimens were designed to exhibit bond (brittle) failure owing to insufficient lap-splice lengths, whereas the remaining six exhibited ductile behavior, failing only after the lap-spliced rebars yielded. The measured corrosion levels were 0.78–3.17% and 4.21–17.96% for the longitudinal rebars and stirrups in the lap-splice region, respectively. A comparison of these test results with 12 non-corroded beams (fabricated in the same batch and tested in our previous study [54]) resulted in the following conclusions:

1. All the corroded beams exhibited higher load-carrying capacities than their non-corroded counterparts. For brittle failure beams, the peak load increase was 8.64–27.68%. Conversely, the value was 0.13–5.77% for the ductile failure beams. These findings can be explained thus: low corrosion causes an increase in surface roughness of rebars and formation of expansive rust particles, which improve the frictional stress and mechanical interlock between rebar and surrounding concrete, thereby improving the bond strength and load-carrying capacity of beams.
2. The stirrups, despite corrosion, provided sufficient confinement to tensile rebars and did not affect the bond performance of lap-splices.
3. The improved bonding between rebar and surrounding concrete due to low corrosion levels increased the initial stiffnesses of the corroded beams more than those of the non-corroded beams. This indicated that the deflections of beams with lower corrosion levels would be less than those of non-corroded beams.
4. Some corroded beams with sufficient lap-splice lengths exhibited decreased ductility index compared with the non-corroded beams of their respective groups. However, residual ductility still sufficiently satisfied the seismic-design requirements.
5. Although most corroded beams with insufficient lap-splice lengths exhibited higher ductility indices than their corresponding non-corroded counterparts (with a few exceptions), these beams still suffered sudden (brittle) failures primarily because of the insufficient lap-splice length rather than the corrosion itself. Therefore, retrofitting should be provided for old RC piers with insufficient lap-splice lengths.
6. Corroded beams with insufficient lap-splice lengths exhibited increased bond strengths of 13–43% compared with the predictions from existing empirical equations, such as those proposed by Orangun et al. [65], Darwin et al. [66], and Esfahani and Kianoush [67]. This variance indicated that these established equations only provided conservative predictions for the bond strengths of lap-splices at low corrosion levels.

## Figures and Tables

**Table 1:** Specimen details [54].

Specimens	Notation	$f_{c}^{\prime }$ MPa	$f_{y}$ MPa	$d_{s}$ mm	N	d mm	$c_{b}$ mm	$c_{so}$ mm	$c_{si}$ mm	$l_{s,test}$ mm	$l_{s,ACI}$ mm
Group 1	C27Y500S10	27.10	500	10	7	348.50	34	62	43	610	1014
Group 2	C27Y500S13	27.50	500	13	6	350.50	32	56	49	610	1006
Group 3	C27Y300S10	26.75	300	10	7	348.50	34	62	43	610	532
Group 4	C24Y300S13	23.45	300	13	6	350.50	32	56	49	610	569

**Table 2:** Corrosion and load capacities of the beams.

Failure Mode	Corroded Specimens					Non-Corroded Specimens			Difference (%)
	Specimen	Rebar Type	$C_{l}$ %	$C_{t}$ %	$P_{C}$ kN	Specimen	$P_{N}$ kN	$P_{N,avg}$	$\frac{P_{C}-P_{N,avg}}{P_{N,avg}}\times 100$
$l_{s,test}$$\;<\;l_{s,ACI}$ Bond (brittle) Failure	CC27Y500S10-1	KSHS	3.17	14.36	108.79	NC27Y500S10-1	104.23	98.46	10.49
	CC27Y500S10-2		3.03	19.08	125.71	NC27Y500S10-2	101.83		27.68
	CC27Y500S10-3		2.34	13.80	106.97	NC27Y500S10-3	89.32		8.64
	CC27Y500S13-1	TWPS	1.01	10.31	112.76	NC27Y500S13-1	91.62	95.71	17.81
	CC27Y500S13-2		0.78	8.77	113.94	NC27Y500S13-2	104.87		19.05
	CC27Y500S13-3		1.02	5.04	113.11	NC27Y500S13-3	90.64		18.18
$l_{s,test}$$\;<\;l_{s,ACI}$ Yielding (ductile) Failure	CC27Y300S10-1	TWPS	0.79	5.66	78.72	NC27Y300S10-1	74.26	74.85	5.17
	CC27Y300S10-2		0.91	11.76	79.17	NC27Y300S10-2	76.27		5.77
	CC27Y300S10-3		1.05	6.44	74.95	NC27Y300S10-3	74.02		0.13
	CC24Y300S13-1	KSHS	3.26	11.46	81.67	NC24Y300S13-1	76.47	77.09	5.94
	CC24Y300S13-2		3.52	4.21	78.18	NC24Y300S13-2	78.78		1.41
	CC24Y300S13-3		1.20	8.82	79.26	NC24Y300S13-3	76.03		2.81

**Table 3:** Initial stiffnesses and ductilities of the beams.

Failure Mode	Specimen	$K_{C}$ kN/mm	$\mu_{C}$	Specimen	$K_{N}$ kN/mm	$\mu_{N}$
Bond (brittle) Failure	CC27Y500S10-1	4.66	1.20	NC27Y500S10-1	3.46	1.14
	CC27Y500S10-2	4.14	1.21	NC27Y500S10-2	3.32	1.17
	CC27Y500S10-3	4.56	1.21	NC27Y500S10-3	3.36	1.18
	CC27Y500S13-1	4.52	1.26	NC27Y500S13-1	3.61	1.20
	CC27Y500S13-2	4.69	1.23	NC27Y500S13-2	3.78	1.16
	CC27Y500S13-3	4.77	1.22	NC27Y500S13-3	3.77	1.35
Yielding (ductile) Failure	CC27Y300S10-1	4.86	10.70	NC27Y300S10-1	3.55	13.50
	CC27Y300S10-2	4.96	5.72	NC27Y300S10-2	3.63	11.01
	CC27Y300S10-3	-	-	NC27Y300S10-3	3.52	9.90
	CC24Y300S13-1	5.46	6.72	NC24Y300S13-1	3.90	9.57
	CC24Y300S13-2	5.17	12.77	NC24Y300S13-2	3.66	11.97
	CC24Y300S13-3	5.22	6.35	NC24Y300S13-3	3.53	11.73

**Table 4:** Bond stress at failure for beams with insufficient lap-splice lengths.

Specimen	P kN	$f_{c}^{\prime }$ MPa	ρ	$E_{C}$ MPa	n	k	j=1−k3	$f_{s}$ MPa	$u^{T}$ MPa
C27Y500S10-1	108.79	27.10	0.009083	24,467.10	8.99	0.33	0.89	500.55	5.13
C27Y500S10-2	125.71	27.10	0.009083	24,467.10	8.99	0.33	0.89	578.40	5.93
C27Y500S10-3	106.97	27.10	0.009083	24,467.10	8.99	0.33	0.89	492.18	5.04
C27Y500S13-1	112.76	27.50	0.009031	24,647.01	8.93	0.33	0.89	515.53	5.28
C27Y500S13-2	113.94	27.50	0.009031	24,647.01	8.93	0.33	0.89	520.92	5.34
C27Y500S13-3	113.11	27.50	0.009031	24,647.01	8.93	0.33	0.89	517.13	5.30

$k=\sqrt{2n\rho {+\left(n\rho \right)}^{2}}-n\rho$, $E_{s}$ = 220,000 MPa, $E_{c}=4700\sqrt{f_{c}^{\prime }}$, n=EsEc, and $A_{S}$= 981.25 mm^2^

**Table 5:** Empirical equations for calculating the bond strengths of lap-splices.

Reference	Equation
Orangun et al. (1975) [67]	$u\;=\left[1.2+3\left(\frac{c}{d_{b}}\right)+50\left(\frac{d_{b}}{l_{s}}\right)+k_{tr}^{\prime }\right]\sqrt{f_{c}^{\prime }}\;\mathrm{for}\;\frac{c}{d_{b}}\;\leq 2.5,\;\mathrm{where}\;k_{tr}^{\prime }=\frac{A_{tr}f_{yt}}{500\;s\;d_{b}}\mathrm{for}\;k_{tr}^{\prime }\;\leq \;3.0\text{ [Imperial units]}$
Darwin et al. (1995) [68]	$u=\frac{{\left(f_{c}^{\prime }\right)}^{\frac{1}{4}}}{\pi d_{b}l_{s}}\left[63l_{d}\left(c+0.5d_{b}\right)+2130A_{b}\right]\left(0.1\frac{c_{M}}{c}+0.9\right)+2226t_{r}t_{d}\frac{NA_{tr}}{n}\;\mathrm{where}\;t_{r}=\;9.6R_{r}\;+\;0.28\;\mathrm{and}\;t_{d}\;=\;0.72d_{b}\;+\;0.28\text{ [Imperial units]}$
Esfahani and Kianoush (2005) [69]	u =uc1+1M1.85+0.024M0.88+0.12CmedCmin ×1+0.015fRAtrAbCswhere uc=2.7Cdb +0.5Cdb +3.6 fc′ and M=cosh0.0022ls3fc′db [SI Units]

$A_{b}$: area of a single lap-splice rebar: $A_{tr}$: area of the transverse reinforcement (single leg of a stirrup); c: minimum of $C_{b}$, $C_{s}$, or $C_{cl}$; $c_{M}$: maximum of $C_{b}$, $C_{s}$, or $C_{cl}$; $C_{med}$: median of $C_{b}$, $C_{s}$, or $C_{cc}$; $C_{min}$: minimum of $C_{b}$, $C_{s}$, or $C_{cc}$; $C_{b}$ and $C_{s}$: bottom and side covers of the lap-spliced rebar, respectively; $C_{cc}$ and $C_{cl}$: center-to-center and clear spacing of the lap-spliced rebars, respectively; $d_{b}$: diameter of lap-spliced rebar; $f_{c}^{\prime }$: 28-day compressive strength of concrete; $f_{R}$ is 1 for $R_{r}$ < 0.11 and 1.6 for $R_{r}$ ≥ 0.11; $f_{yt}$: yield strength of transverse reinforcement; $l_{s}$: lap-splice length; N: number of stirrups in the lap-splice region; n: number of lap-spliced rebars; $R_{r}$: relative rib area of the lap-spliced rebar; and s: stirrup spacing.

**Table 6:** Bond strength of lap-splices.

Specimen (Corroded)	Corrosion Level (%)	Bond Strength (MPa)	Specimen (Non-Corroded)	Bond Strength (MPa)	Bond Strength (Percentage Increase in Bond Strength of Corroded Lap-Splices)			
					Average Non-Corroded	Orangun et al. (1975) [67]	Darwin et al. (1995) [68]	Esfahani & Kianoush (2005) [69]
CC27Y500S10-1	3.17	5.13	NC27Y500S10-1	5.07	4.79(7.17)	4.46(15.02)	4.15(23.61)	4.08(25.74)
CC27Y500S10-2	3.03	5.93	NC27Y500S10-2	4.95	4.79(23.89)	4.46(32.96)	4.15(42.89)	4.08(45.34)
CC27Y500S13-3	2.34	5.04	NC27Y500S13-3	4.34	4.79(5.29)	4.46(13.00)	4.15(21.45)	4.08(23.53)
CC27Y300S10-1	1.01	5.28	NC27Y300S10-1	4.43	4.63(14.12)	4.39(20.27)	4.22(25.12)	4.33(21.94)
CC24Y300S10-2	0.78	5.34	NC24Y300S10-2	5.07	4.63(15.42)	4.39(21.64)	4.22(26.54)	4.33(23.33)
CC24Y300S10-3	1.02	5.30	NC24Y300S10-3	4.38	4.63(14.55)	4.39(20.73)	4.22(25.59)	4.33(22.40)

**Table 7:** Stress slopes of the beams with insufficient lap-splice lengths.

Beam	Loading Stage	Slope of Corroded Rebar Stress				Slope of Non-Corroded Rebar Stress			
		FO	FI	BI	BO	FO	FI	BI	BO
C27Y500S10-1	A	0.0642	−0.0979	0.2125	−0.1776	0.0946	−0.1064	0.1483	−0.0618 *
	B	0.5942	−0.6575	0.6444	−0.6200	0.6384	−0.7504	0.7941	−0.6247 *
	C	0.9279	−1.1107	1.0794	−0.9589	1.0511	−1.1701	1.2811	−0.981 *
	D	0.6567	−0.7455	0.3796	−0.7507	0.7666	−0.8615	0.682	−0.7713 *
C27Y500S13-1	A	0.0434	−0.0397	0.0502	−0.0409	0.0778	−0.1592	0.0862	−0.1863
	B	0.7510	−0.7008	0.7172	−0.7741	0.6936	−0.6417	0.6386	−0.7496
	C	1.1629	−1.079	0.9513	−1.3474	1.0247	−0.9011	0.9208	−1.0840
	D	0.7555	−0.5207	0.9957	−1.0539	0.8068	−1.0006	0.8194	−0.7835

* Since one of the strain gauges of the rebar was non-operational, three values were used for the linear regression, whereas the others used four.

## Data Availability

The original contributions presented in this study are included in the article. Further inquiries can be directed to the corresponding author.
